# Methyl 3-[(*E*)-(2-hy­droxy-1-naphth­yl)methyl­idene]carbazate

**DOI:** 10.1107/S1600536810044041

**Published:** 2010-11-06

**Authors:** Liang-Quan Sheng, Hua-Jie Xu, Na-Na Du, Xue-Yue Jiang

**Affiliations:** a Department of Chemistry, Fuyang Normal College, Fuyang, Anhui 236041, People’s Republic of China

## Abstract

The title compound, C_13_H_12_N_2_O_3_, has an *E* configuration with respect to the C=N bond: the conformation is stabilized by an intramolecular O—H⋯N hydrogen bond. In the crystal, an N—H⋯O interaction links the molecules into a *C*(4) chain along [100].

## Related literature

For the naphthalene group as a fluoro­phore, see: Li *et al.* (2010 ▶); Iijima *et al.* (2010 ▶). For a related structure and bond length, see: Xu *et al.* (2009 ▶). For the synthetic method, see: Zhang *et al.* (1999 ▶). For graph-set notation, see: Bernstein *et al.* (1995 ▶). For applications of Schiff base–metal complexes, see: Cozzi (2004 ▶).
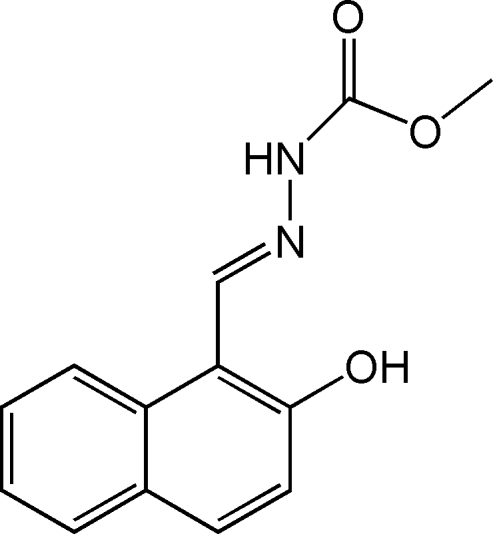


## Experimental

### 

#### Crystal data

C_13_H_12_N_2_O_3_
*M* *_r_* = 244.25
Orthorhombic, 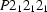

*a* = 5.1754 (3) Å
*b* = 9.2787 (5) Å
*c* = 23.6766 (12) Å
*V* = 1136.97 (11) Å^3^
*Z* = 4
Mo *K*α radiation
μ = 0.10 mm^−1^
*T* = 291 K
0.40 × 0.36 × 0.30 mm

#### Data collection

Oxford Diffraction Gemini S Ultra diffractometer
Absorption correction: multi-scan (*CrysAlis PRO*; Oxford Diffraction, 2009 ▶) *T*_min_ = 0.960, *T*_max_ = 0.970
6329 measured reflections
1562 independent reflections
1120 reflections with *I* > 2σ(*I*)
*R*_int_ = 0.031

#### Refinement

*R*[*F* ^2^ > 2σ(*F* ^2^)] = 0.030
*wR*(*F* ^2^) = 0.058
*S* = 1.09
1562 reflections
165 parameters
H-atom parameters constrained
Δρ_max_ = 0.13 e Å^−3^
Δρ_min_ = −0.13 e Å^−3^

### 

Data collection: *CrysAlis PRO* (Oxford Diffraction, 2009 ▶); cell refinement: *CrysAlis PRO*; data reduction: *CrysAlis PRO*; program(s) used to solve structure: *SHELXS97* (Sheldrick, 2008 ▶); program(s) used to refine structure: *SHELXL97* (Sheldrick, 2008 ▶); molecular graphics: *ORTEP-3* (Farrugia, 1997 ▶); software used to prepare material for publication: *SHELXL97*.

## Supplementary Material

Crystal structure: contains datablocks global, I. DOI: 10.1107/S1600536810044041/bx2318sup1.cif

Structure factors: contains datablocks I. DOI: 10.1107/S1600536810044041/bx2318Isup2.hkl

Additional supplementary materials:  crystallographic information; 3D view; checkCIF report

## Figures and Tables

**Table 1:** Hydrogen-bond geometry (Å, °)

*D*—H⋯*A*	*D*—H	H⋯*A*	*D*⋯*A*	*D*—H⋯*A*
O1—H1⋯N1	0.82	1.91	2.6332 (18)	146
N2—H2⋯O2^i^	0.86	2.11	2.9626 (18)	170

Symmetry code: (i) 
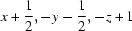
.

**Fig. 1.**
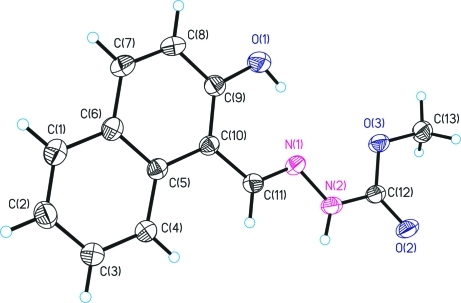
The molecular structure of the title compound, showing 30% probability displacement ellipsoids.

**Fig. 2.**
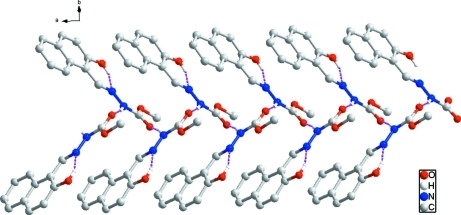
The one-dimensional structure of the title compound formed by intermolecular hydrogen bonds viewed along the c axis. The intra- and intermolecular hydrogen bonds are shown as pink dashed lines.

## supplementary crystallographic information

### Comment

The naphthalene group as a fluorophore has been studied extensively due to its characteristic photophysical properties and the competitive stability in the environment (Li *et al.*, 2010; Iijima *et al.*, 2010). Schiff base metal complexes have been widely studied because they have industrial, antifungal, antibacterial, anticancer and herbicidal applications (Cozzi, 2004). As part of an ongoing study of Schiff bases incorporating the naphthalene group (Xu *et al.*, 2009), we report here on the crystal structure of the title compound.

The molecular structure of the title compound is shown in Fig. 1 and geometrical parameters are given in the archived CIF. The title molecule, adopts an *E* or *trans* configuration with respect to the C=N bond while the lengths and angles are within normal ranges. The C=N bond length is 1.288 (2) Å, a little longer than schiff base C=N bond length (1.280 (15) Å) (Xu *et al.*, 2009).The crystal structure of (I) is stabilized by one intramolecular O—H···N interaction with H···N distances 1.91Å and O—H···N angles is 146.1° and one intermolecular N—H···O interaction with H···O distances 2.11Å and N—H···O angles is 169.8°, Table 1. The molecules are linked in C(4) chains along [100], (Bernstein *et al.*, 1995), Fig. 2.

### Experimental

All reagents and solvents were used as obtained commercially without further purification. The title compound was prepared according to the reported procedure (Zhang *et al.*, 1999). A solution of methyl carbazate (0.09 g, 1 mmol) in 5 ml of ethanol was added slowly to a solution of 2-hydro-1-naphthaldehyde (0.172 g, 1 mmol) in 15 ml absolute ethanol, under heating and stirring. The mixture was refluxed for 3 h, then cooled to room temperature and left to stand in air for 5 days. Yellow block-shaped crystals were formed on slow evaporation of the solvent.

### Refinement

H atoms bonded to C atoms were placed geometrically and treated as riding, with C—H distances 0.93–0.96 Å and *U*_iso_(H) = 1.2*U*_eq_(C) for the CH, while *U*_iso_(H) = 1.5*U*_eq_(C) for the CH_3_ groups. The amide H atoms were located from difference maps and refined with the N—H distances restrained to 0.86 Å and *U*_iso_(H) = 1.2*U*_eq_(N). The hydroxyl H atoms were located from difference maps and refined with the O—H distances restrained to 0.82 Å and *U*_iso_(H) = 1.5*U*_eq_(O).

### Crystal data

**Table:**

C_13_H_12_N_2_O_3_	*F*(000) = 512
*M**_r_* = 244.25	*D*_x_ = 1.427 Mg m^−^^3^
Orthorhombic, *P*2_1_2_1_2_1_	Mo *K*α radiation, λ = 0.71073 Å
Hall symbol: P 2ac 2ab	Cell parameters from 2986 reflections
*a* = 5.1754 (3) Å	θ = 3.4–29.1°
*b* = 9.2787 (5) Å	µ = 0.10 mm^−^^1^
*c* = 23.6766 (12) Å	*T* = 291 K
*V* = 1136.97 (11) Å^3^	Block, yellow
*Z* = 4	0.40 × 0.36 × 0.30 mm

### Data collection

**Table:**

Oxford Diffraction Gemini S Ultra diffractometer	1562 independent reflections
Radiation source: Enhance (Mo) X-ray Source	1120 reflections with *I* > 2σ(*I*)
graphite	*R*_int_ = 0.031
Detector resolution: 15.9149 pixels mm^-1^	θ_max_ = 27.9°, θ_min_ = 3.4°
ω scans	*h* = −6→6
Absorption correction: multi-scan (*CrysAlis PRO*; Oxford Diffraction, 2009)	*k* = −12→12
*T*_min_ = 0.960, *T*_max_ = 0.970	*l* = −30→29
6329 measured reflections	

### Refinement

**Table:**

Refinement on *F*^2^	Primary atom site location: structure-invariant direct methods
Least-squares matrix: full	Secondary atom site location: difference Fourier map
*R*[*F*^2^ > 2σ(*F*^2^)] = 0.030	Hydrogen site location: inferred from neighbouring sites
*wR*(*F*^2^) = 0.058	H-atom parameters constrained
*S* = 1.09	*w* = 1/[σ^2^(*F*_o_^2^) + (0.0239*P*)^2^] where *P* = (*F*_o_^2^ + 2*F*_c_^2^)/3
1562 reflections	(Δ/σ)_max_ < 0.001
165 parameters	Δρ_max_ = 0.13 e Å^−^^3^
0 restraints	Δρ_min_ = −0.13 e Å^−^^3^

### Special details

**Table:**

Experimental. Absorption correction: CrysAlisPro,(Oxford Diffraction 2009). Version 1.171.33.66 (release 28-04-2010 CrysAlis171 .NET) (compiled Apr 28 2010,14:27:37) Empirical absorption correction using spherical harmonics, implemented in SCALE3 ABSPACK scaling algorithm.
Geometry. All e.s.d.'s (except the e.s.d. in the dihedral angle between two l.s. planes) are estimated using the full covariance matrix. The cell e.s.d.'s are taken into account individually in the estimation of e.s.d.'s in distances, angles and torsion angles; correlations between e.s.d.'s in cell parameters are only used when they are defined by crystal symmetry. An approximate (isotropic) treatment of cell e.s.d.'s is used for estimating e.s.d.'s involving l.s. planes.
Refinement. Refinement of *F*^2^ against ALL reflections. The weighted *R*-factor *wR* and goodness of fit *S* are based on *F*^2^, conventional *R*-factors *R* are based on *F*, with *F* set to zero for negative *F*^2^. The threshold expression of *F*^2^ > σ(*F*^2^) is used only for calculating *R*-factors(gt) *etc*. and is not relevant to the choice of reflections for refinement. *R*-factors based on *F*^2^ are statistically about twice as large as those based on *F*, and *R*- factors based on ALL data will be even larger.

### Fractional atomic coordinates and isotropic or equivalent isotropic displacement parameters (Å^2^)

**Table:**

	*x*	*y*	*z*	*U*_iso_*/*U*_eq_	
C1	0.7358 (4)	0.2360 (2)	0.64442 (8)	0.0492 (5)	
H1A	0.8342	0.2851	0.6711	0.059*	
C2	0.7869 (4)	0.2539 (2)	0.58880 (8)	0.0538 (5)	
H2A	0.9175	0.3163	0.5774	0.065*	
C3	0.6428 (4)	0.1785 (2)	0.54858 (8)	0.0529 (5)	
H3	0.6800	0.1901	0.5104	0.064*	
C4	0.4483 (4)	0.0881 (2)	0.56460 (7)	0.0460 (5)	
H4	0.3539	0.0397	0.5371	0.055*	
C5	0.3871 (3)	0.06633 (18)	0.62220 (7)	0.0363 (4)	
C6	0.5348 (3)	0.14347 (18)	0.66256 (7)	0.0402 (4)	
C7	0.4774 (4)	0.1259 (2)	0.72052 (7)	0.0478 (5)	
H7	0.5710	0.1780	0.7471	0.057*	
C8	0.2899 (4)	0.0355 (2)	0.73821 (7)	0.0476 (5)	
H8	0.2564	0.0256	0.7766	0.057*	
C9	0.1459 (3)	−0.04352 (19)	0.69896 (7)	0.0406 (4)	
C10	0.1867 (3)	−0.02866 (18)	0.64118 (7)	0.0354 (4)	
C11	0.0239 (3)	−0.10114 (18)	0.60072 (6)	0.0396 (4)	
H11	0.0471	−0.0813	0.5626	0.047*	
C12	−0.4938 (4)	−0.33819 (19)	0.57803 (6)	0.0397 (4)	
C13	−0.7307 (4)	−0.4828 (2)	0.64025 (8)	0.0554 (5)	
H13A	−0.8917	−0.4395	0.6294	0.083*	
H13B	−0.7372	−0.5087	0.6795	0.083*	
H13C	−0.7014	−0.5677	0.6179	0.083*	
N1	−0.1522 (3)	−0.19204 (16)	0.61512 (6)	0.0410 (4)	
N2	−0.2962 (3)	−0.24594 (17)	0.57083 (5)	0.0455 (4)	
H2	−0.2578	−0.2193	0.5371	0.055*	
O1	−0.0360 (3)	−0.13205 (14)	0.72081 (5)	0.0553 (4)	
H1	−0.1165	−0.1705	0.6951	0.083*	
O2	−0.6293 (2)	−0.37715 (15)	0.53872 (5)	0.0548 (4)	
O3	−0.5234 (2)	−0.38178 (12)	0.63113 (4)	0.0462 (3)	

### Atomic displacement parameters (Å^2^)

**Table:**

	*U*^11^	*U*^22^	*U*^33^	*U*^12^	*U*^13^	*U*^23^
C1	0.0448 (11)	0.0476 (11)	0.0551 (11)	−0.0007 (12)	−0.0054 (9)	−0.0056 (10)
C2	0.0448 (11)	0.0570 (12)	0.0597 (12)	−0.0065 (12)	0.0032 (11)	0.0074 (11)
C3	0.0487 (12)	0.0657 (14)	0.0443 (10)	−0.0078 (12)	−0.0017 (10)	0.0070 (10)
C4	0.0413 (11)	0.0562 (12)	0.0404 (9)	−0.0019 (11)	−0.0027 (9)	0.0043 (9)
C5	0.0347 (9)	0.0353 (9)	0.0388 (9)	0.0067 (9)	−0.0037 (8)	0.0020 (8)
C6	0.0385 (10)	0.0368 (10)	0.0453 (9)	0.0044 (11)	−0.0051 (9)	0.0009 (9)
C7	0.0546 (11)	0.0499 (11)	0.0390 (9)	−0.0025 (13)	−0.0065 (10)	−0.0068 (9)
C8	0.0570 (12)	0.0542 (12)	0.0315 (9)	0.0008 (12)	−0.0029 (9)	−0.0013 (9)
C9	0.0406 (10)	0.0410 (10)	0.0404 (10)	0.0028 (10)	−0.0001 (9)	0.0058 (9)
C10	0.0359 (10)	0.0358 (9)	0.0346 (9)	0.0044 (10)	−0.0040 (8)	0.0002 (8)
C11	0.0401 (10)	0.0427 (10)	0.0359 (9)	0.0007 (12)	0.0000 (8)	0.0025 (8)
C12	0.0427 (10)	0.0428 (10)	0.0336 (9)	0.0023 (11)	−0.0029 (9)	−0.0007 (8)
C13	0.0492 (12)	0.0622 (12)	0.0547 (11)	−0.0121 (13)	−0.0041 (10)	0.0117 (10)
N1	0.0422 (9)	0.0446 (9)	0.0362 (7)	−0.0021 (9)	−0.0076 (7)	−0.0017 (7)
N2	0.0503 (9)	0.0556 (9)	0.0305 (7)	−0.0110 (10)	−0.0022 (7)	0.0015 (7)
O1	0.0580 (8)	0.0671 (9)	0.0406 (6)	−0.0135 (9)	0.0007 (7)	0.0059 (7)
O2	0.0601 (8)	0.0684 (9)	0.0360 (6)	−0.0118 (9)	−0.0126 (6)	0.0004 (7)
O3	0.0506 (7)	0.0528 (7)	0.0352 (6)	−0.0126 (8)	−0.0053 (6)	0.0075 (6)

### Geometric parameters (Å, °)

**Table:**

C1—C2	1.353 (2)	C9—O1	1.3523 (19)
C1—C6	1.416 (2)	C9—C10	1.391 (2)
C1—H1A	0.9300	C10—C11	1.442 (2)
C2—C3	1.397 (3)	C11—N1	1.288 (2)
C2—H2A	0.9300	C11—H11	0.9300
C3—C4	1.364 (2)	C12—O2	1.2203 (19)
C3—H3	0.9300	C12—O3	1.3294 (19)
C4—C5	1.414 (2)	C12—N2	1.344 (2)
C4—H4	0.9300	C13—O3	1.441 (2)
C5—C6	1.418 (2)	C13—H13A	0.9600
C5—C10	1.433 (2)	C13—H13B	0.9600
C6—C7	1.413 (2)	C13—H13C	0.9600
C7—C8	1.350 (2)	N1—N2	1.3804 (18)
C7—H7	0.9300	N2—H2	0.8600
C8—C9	1.399 (2)	O1—H1	0.8200
C8—H8	0.9300		
			
C2—C1—C6	120.88 (18)	O1—C9—C10	122.83 (16)
C2—C1—H1A	119.6	O1—C9—C8	115.79 (15)
C6—C1—H1A	119.6	C10—C9—C8	121.38 (17)
C1—C2—C3	119.82 (19)	C9—C10—C5	118.63 (16)
C1—C2—H2A	120.1	C9—C10—C11	121.23 (16)
C3—C2—H2A	120.1	C5—C10—C11	120.08 (14)
C4—C3—C2	120.82 (17)	N1—C11—C10	122.89 (14)
C4—C3—H3	119.6	N1—C11—H11	118.6
C2—C3—H3	119.6	C10—C11—H11	118.6
C3—C4—C5	121.39 (17)	O2—C12—O3	124.41 (18)
C3—C4—H4	119.3	O2—C12—N2	121.93 (15)
C5—C4—H4	119.3	O3—C12—N2	113.66 (15)
C4—C5—C6	117.21 (16)	O3—C13—H13A	109.5
C4—C5—C10	123.50 (16)	O3—C13—H13B	109.5
C6—C5—C10	119.29 (15)	H13A—C13—H13B	109.5
C7—C6—C1	121.25 (17)	O3—C13—H13C	109.5
C7—C6—C5	118.88 (17)	H13A—C13—H13C	109.5
C1—C6—C5	119.87 (15)	H13B—C13—H13C	109.5
C8—C7—C6	121.60 (17)	C11—N1—N2	114.73 (13)
C8—C7—H7	119.2	C12—N2—N1	123.02 (13)
C6—C7—H7	119.2	C12—N2—H2	118.5
C7—C8—C9	120.18 (16)	N1—N2—H2	118.5
C7—C8—H8	119.9	C9—O1—H1	109.5
C9—C8—H8	119.9	C12—O3—C13	115.17 (14)
			
C6—C1—C2—C3	−1.1 (3)	C5—C10—C9—O1	−179.05 (14)
C1—C2—C3—C4	0.9 (3)	C5—C10—C9—C8	2.1 (2)
C5—C4—C3—C2	−0.6 (3)	C11—C10—C9—O1	4.0 (3)
C6—C5—C4—C3	0.6 (3)	C11—C10—C9—C8	−174.82 (16)
C10—C5—C4—C3	−179.27 (16)	C9—C10—C5—C4	178.98 (18)
C2—C1—C6—C7	−178.94 (18)	C9—C10—C5—C6	−0.9 (2)
C2—C1—C6—C5	1.1 (3)	C11—C10—C5—C4	−4.0 (2)
C4—C5—C6—C7	179.26 (17)	C11—C10—C5—C6	176.13 (15)
C10—C5—C6—C7	−0.9 (2)	C9—C10—C11—N1	−6.0 (2)
C4—C5—C6—C1	−0.8 (2)	C5—C10—C11—N1	177.12 (14)
C10—C5—C6—C1	179.04 (15)	N2—N1—C11—C10	177.35 (15)
C1—C6—C7—C8	−178.46 (16)	N1—N2—C12—O2	175.09 (16)
C5—C6—C7—C8	1.5 (3)	N1—N2—C12—O3	−5.3 (2)
C9—C8—C7—C6	−0.2 (3)	C12—N2—N1—C11	−177.71 (15)
C7—C8—C9—O1	179.49 (16)	C13—O3—C12—O2	0.6 (3)
C7—C8—C9—C10	−1.6 (3)	C13—O3—C12—N2	−178.95 (13)

### Hydrogen-bond geometry (Å, °)

**Table:**

*D*—H···*A*	*D*—H	H···*A*	*D*···*A*	*D*—H···*A*
O1—H1···N1	0.82	1.91	2.6332 (18)	146
N2—H2···O2^i^	0.86	2.11	2.9626 (18)	170

Symmetry codes: (i) *x*+1/2, −*y*−1/2, −*z*+1.
